# Supplementary material for: Analysis of PPARγ Signaling Activity in Psoriasis
Source: Int J Mol Sci. 2021 Aug 10;22(16):8603. doi: 10.3390/ijms22168603 (PMC8395241; doi:10.3390/ijms22168603)
Supplement: Supplementary file 1 [file ijms-22-08603-s001.zip › Supplemental materials_Analysis of PPARg signaling activity in psoriasis/Pathway models/Models images and html files/PPARG inhibits phenotypes assotiated with psoriasis/142350.html]

T-helper cell response


# Cell Process T-helper cell response

|  |  |
| --- | --- |
| URN | urn:agi-cellproc:T-helper%20lymphocyte%20response |
| Total Entities | 0 |
| Connectivity | 897 |
| Name | T-helper cell response |
| ObjectType | Cell Process |

---

|  |  |
| --- | --- |
| Pathway | NOD-like Receptors |
|  | Dendritic Cells Function in Atherosclerosis |
|  | Apoptotic Keratinocytes Clearance Recession in Systemic Lupus Erythematosus |
|  | Dendritic Cell Activation in Systemic Lupus Erythematosis |
|  | Th1-Cell Function in Systemic Lupus Erythematosus |
|  | Conventional Dendritic Cell Role in Diabetes Mellitus Type 1 |
|  | e |
|  | PPARG inhibits phenotypes assotiated with psoriasis |
|  | efects selected |
|  | dsRNA induces innate immunity and INFs in alveolar cells |
|  | Dendritic Cell Activation in COVID19 |
|  | Figure 4\_Diabetes mellitus Type 1: conventional dendritic cell |

---

|  |  |
| --- | --- |
| MedScan ID | 4000212 |

---

|  |  |
| --- | --- |
| Alias | type 2 helper cell responses |
|  | T-helper 1 responses |
|  | helper T lymphocyte type 17 responses |
|  | T-helper cell response |
|  | Th17 cell responses |
|  | helper T lymphocyte type 1 responses |
|  | T-helper 2 responses |
|  | th2 lymphocyte responses |
|  | helper T lymphocyte type 2 responses |
|  | helper-inducer T-lymphocyte responses |
|  | Th2 cell responses |
|  | T helper responses |
|  | T inducer cell responses |
|  | T-helper cell responses |
|  | th1 lymphocyte responses |
|  | T inducer lymphocyte responses |
|  | type 17 helper cell responses |
|  | T-helper lymphocyte responses |
|  | Th1 cell responses |
|  | th17 lymphocyte responses |
|  | helper T lymphocyte responses |
|  | type 1 helper cell responses |
|  | T-helper 17 responses |
|  | T helper cell responses |

---
